# Hydrogen Sulfide Protects against Paraquat-Induced Acute Liver Injury in Rats by Regulating Oxidative Stress, Mitochondrial Function, and Inflammation

**DOI:** 10.1155/2020/6325378

**Published:** 2020-01-23

**Authors:** Zhenning Liu, Xiaofeng Wang, Lei Li, Guigui Wei, Min Zhao

**Affiliations:** Department of Emergency Medicine, Shengjing Hospital of China Medical University, No. 36, Sanhao Street, Heping District, Shenyang, Liaoning 110004, China

## Abstract

In addition to the lung, the liver is considered another major target for paraquat (PQ) poisoning. Hydrogen sulfide (H_2_S) has been demonstrated to be effective in the inhibition of oxidative stress and inflammation. The aim of this study was to investigate the protective effect of exogenous H_2_S against PQ-induced acute liver injury. The acute liver injury model was established by a single intraperitoneal injection of PQ, evidenced by histological alteration and elevated serum aminotransferase levels. Different doses of NaHS were administered intraperitoneally one hour before exposure to PQ. Analysis of the data shows that exogenous H_2_S attenuated the PQ-induced liver injury and oxidative stress in a dose-dependent manner. H_2_S significantly suppressed reactive oxygen species (ROS) generation and the elevation of malondialdehyde content while it increased the ratio of GSH/GSSG and levels of antioxidant enzymes including SOD, GSH-Px, HO-1, and NQO-1. When hepatocytes were subjected to PQ-induced oxidative stress, H_2_S markedly enhanced nuclear translocation of Nrf2 via S-sulfhydration of Keap1 and resulted in the increase in IDH2 activity by regulating S-sulfhydration of SIRT3. In addition, H_2_S significantly suppressed NLRP3 inflammasome activation and subsequent IL-1*β* excretion in PQ-induced acute liver injury. Moreover, H_2_S cannot reverse the decrease in SIRT3 and activation of the NLRP3 inflammasome caused by PQ in Nrf2-knockdown hepatocytes. In summary, H_2_S attenuated the PQ-induced acute liver injury by enhancing antioxidative capability, regulating mitochondrial function, and suppressing ROS-induced NLRP3 inflammasome activation. The antioxidative effect of H_2_S in PQ-induced liver injury can at least partly be attributed to the promotion of Nrf2-driven antioxidant enzymes via Keap1 S-sulfhydration and regulation of SIRT3/IDH2 signaling via Nrf2-dependent SIRT3 gene transcription as well as SIRT3 S-sulfhydration. Thus, H_2_S supplementation can form the basis for a promising novel therapeutic strategy for PQ-induced acute liver injury.

## 1. Introduction

Paraquat (PQ) poisoning is a serious clinical problem in developing countries, especially in Asia, since the time it was first applied in agricultural production several decades ago. Due to the lack of specific antidotes and effective treatment methods, acute poisonings from accidental or suicidal ingestion of PQ cause high mortality. Oxidative stress and reactive oxygen species- (ROS-) mediated inflammation are considered the major causes of PQ poisoning [1]. The lung is commonly considered the major target due to the highly developed polyamine uptake system in the alveolar epithelial cells [1]. Nevertheless, the liver is the main source of intrinsic antioxidants that play an important role in enzymatic metabolism and detoxification. Therefore, the liver is more vulnerable to ROS-mediated injury. Previous studies show that PQ intoxication results in acute liver injury characterized by persistent elevation of liver aminotransferases and histopathological changes [2–4]. Clinical data indicates that almost half of PQ-poisoned patients suffer from hepatic complications [5]. Even so, the potential mechanism underlying the pathogenesis of PQ-induced liver injury is still poorly understood.

The Kelch-like ECH-associated protein 1 (Keap1)/nuclear factor erythroid-2-related factor 2 (Nrf2) system is a key regulator of the cellular response to oxidative stress [6]. Under unstressed conditions, Keap1 binds to Nrf2, thereby mediating Nrf2 proteasomal degradation and ubiquitination [7]. Oxidative stress can induce the nuclear accumulation of Nrf2 which can upregulate downstream antioxidant gene transcription thereby promoting the expression of antioxidant enzymes including catalase, superoxide dismutase (SOD), heme oxygenase-1 (HO-1), and glutathione-S-transferase (GST) [8, 9]. The Keap1-Nrf2 system plays an important role in the amelioration of oxidative stress. Based on our previous study [10], the Nrf2-mediated antioxidant system was involved in PQ-induced lung injury resulting in the upregulation of the antioxidant enzyme SOD.

It is widely accepted that persistent redox cycling of PQ results in the continued depletion of nicotinamide adenine dinucleotide phosphate (NADPH) and ROS generation [1]. Sirtuin 3 (SIRT3), the main NAD^+^-dependent deacetylase, has a vital role in the regulation of mitochondrial function and ROS production [11]. Isocitrate dehydrogenase (IDH) is a digestive enzyme which can catalyze the oxidative decarboxylation of isocitrate into alpha-ketoglutarate and produce NADPH to inhibit ROS-mediated cell injury [12, 13]. SIRT3 stimulates the activity of isocitrate dehydrogenase 2 (IDH2) via the deacetylation of IDH2 at lysine 413 [13]. Exposure of endothelial cells to H_2_O_2_ reduces the expression of SIRT3 and IDH2 [14]. Increased SIRT3 expression protects cells against oxidative stress through IDH2 activation [13]. Herein, SIRT3/IDH2 signaling may regulate the mitochondrial redox status. To date, there are no studies directly linking SIRT3/IDH2 signaling to PQ-induced liver injury.

The NLRP3 (*nucleotide-binding domain and leucine-rich repeat containing protein 3*) inflammasome mediates the inflammatory response to various exogenous and endogenous signals including bacterial toxins [15], ATP [15], and ROS [16]. Once the NLRP3 inflammasome is activated, interleukin-1*β* (IL-1*β*) and interleukin-18 (IL-18) are cleaved by active caspase-1 to be released in the active form (IL-1*β* and IL-18) thereby amplifying the inflammatory response [17]. Based on our previous studies [18, 19], PQ activated the NLRP3 inflammasome, leading to proinflammatory cytokine (IL-1*β* and IL-18) secretion in macrophages. Therefore, reduction of oxidative stress and inhibition of the inflammatory response would likely be beneficial in the treatment of PQ poisoning.

Hydrogen sulfide (H_2_S) has been recently considered the third endogenous gasotransmitter next to nitric oxide (NO) and carbon monoxide (CO) [20]. The efficacy of H_2_S mainly involves attenuation of oxidative stress, regulation of mitochondrial function, reduction of lipid peroxidation and inflammatory mediators, and inhibition of apoptosis [21–23]. H_2_S is an important modulator involved in the treatment of DSS-induced colitis [24], attenuation of Alzheimer's disease [25], protection of myocardial ischemia-reperfusion injury [26], and gastroprotection mediated by the gut-brain axis [27, 28]. In addition, H_2_S exerts a protective effect against hepatic ischemia/reperfusion injury, liver cirrhosis, CCl_4_-induced hepatotoxicity, and nonalcoholic steatohepatitis [29–32]. Nonetheless, it remains unclear whether H_2_S has a protective effect against acute liver injury induced by PQ. Thus, the main aim of this study is to investigate the effect of H_2_S on PQ-induced liver injury and to further explore its potential molecular mechanisms: regulation of the Keap1/Nrf2 pathway and SIRT3/IDH2 signaling as well as inhibition of NLRP3 inflammasome activation.

## 2. Materials and Methods

### 2.1. Animals

Healthy male Wistar rats (230 ± 20 g) from the Experimental Animal Center of China Medical University were maintained in standard cages in a temperature- (22 ± 2°C) and humidity-controlled (50% ± 10%) environment with a daily light-dark cycle. The rats had ad libitum access to food and water. All animal experiments were conducted according to the ethical standards of the Institutional Animal Ethics Committee and Animal Care Guidelines of China Medical University.

### 2.2. Chemicals and Reagents

PQ and sodium hydrosulfide (NaHS, a common H_2_S donor) were purchased from Sigma-Aldrich Chemical Corp. (St. Louis, MO, USA). NaHS working solution was prepared 30 min before use. A bicinchoninic acid (BCA) reagent was obtained from Generay Biotech (Shanghai, China). All reagents were of analytical grade.

### 2.3. Experimental Protocols

The rats (*n* = 90) were randomly distributed into five groups (18 rats per group) by using Excel's RANDBETWEEN function: (1) control group (1 mL normal saline solution, i.p.), (2) NaHS-alone group (5 mg/kg, i.p.), (3) PQ group, (4) PQ+NaHS-L group (3 mg/kg, i.p.), and (5) PQ+NaHS-H group (5 mg/kg, i.p.). PQ was intraperitoneally administered in rats at a dose of 20 mg/kg which was based on our previous study [10]. With reference to the previous studies [33–35], two different doses of NaHS as mentioned above were administered intraperitoneally one hour prior to exposure to PQ. At 12 h, 24 h, and 48 h after PQ administration, the rats (*n* = 6) were taken from each group and sacrificed after deep anesthesia at each time point. The liver and blood samples were collected and processed for immunohistochemical and molecular analysis.

### 2.4. Serum Levels of Hepatic Markers

Measurements of serum levels of alanine aminotransferase (ALT) and aspartate aminotransferase (AST) were performed by using the automatic biochemical analyzer (Toshiba, Japan). In brief, the collected blood samples from rats were centrifuged at 3000 rpm for 15 min at 4°C to obtain the serum. The separated serum was then stored at −80°C until used. The bubble-free serum samples (200 *μ*L) were transferred to sample cups in the sample drawer of the automatic biochemical analyzer. At the same time, the slides for detection of ALT and AST, pipette tips, and any other necessary materials were also loaded into the sample drawer. The analyzer was immediately started after the sample drawer was closed. The results were displayed on the screen within 15 min.

### 2.5. H&E Staining and Histopathological Score

Rat liver tissue samples from each study group were fixed in 4% paraformaldehyde for 48 h at 4°C, prior to being embedded in paraffin. According to the routine staining methods, paraffin sections (4 *μ*m thick) were stained with hematoxylin and eosin (H&E). To evaluate the severity of PQ-induced liver injury, histological examination under a microscope and scoring were performed in a double-blinded manner by independent investigators. According to the histopathology scoring described by Suzuki et al. [36], sinusoidal congestion, hepatocyte cytoplasm vacuolization, and parenchymal necrosis were assessed. Histopathology scoring was determined on a scale from 0 to 4 as follows: score 0: none, score 1: minimal/single-cell necrosis, score 2: mild/less than 30% necrosis, score 3: moderate/30%-60% necrosis, and score 4: severe/more than 60% necrosis.

### 2.6. Measurement of ROS Production

ROS production in liver tissue sections was detected by using the ROS Fluorescent Probe-DHE (dihydroethidium) assay kit (Vigorous Biotechnology Beijing Co., Ltd., Beijing, China). In brief, fresh frozen liver tissue sections were incubated with DHE (10 *μ*M) at 37°C for 30 min. After DHE enters the cells, it can be oxidized to yield ethidium and produce bright red fluorescence. After washing with PBS, fluorescence was observed with a fluorescence inverted microscope, using excitation and emission wavelengths of 535 nm and 610 nm, respectively. Quantification of ROS production was evaluated using the Image-Pro Plus software.

### 2.7. Measurement of Oxidative Stress Markers

Rat livers were homogenized separately in 1 mL of 0.05 M Trizma base buffer (pH 7.4) in a glass homogenizer. The homogenates were centrifuged at 14000 × *g* for 20 min at 4°C to obtain a supernatant for subsequent assays. Protein concentrations were determined using the classical Bradford method with bovine serum albumin (BSA) as the standard protein. The malondialdehyde (MDA) content, superoxide dismutase (SOD) activity, glutathione peroxidase (GSH-Px) activity, reduced glutathione (GSH) content, and oxidized glutathione (GSSG) content in the liver were measured with the MDA assay kit (TBA method), SOD assay kit (hydroxylamine method), GSH-PX assay kit (colorimetric method), and total GSH/GSSG assay kit (colorimetric method), respectively. Reduced GSH (nmol/mg protein) was calculated as total GSH − 2 × GSSG. These commercially available kits were purchased from Nanjing Jiancheng Corp. (Nanjing, China). In addition, heme oxygenase 1 (HO-1) and NAD(P)H: quinone oxidoreductase 1 (NQO-1) contents in rat liver tissue were determined by using the rat HO-1 (ab213968) and NQO-1 (ab34173) ELISA kits (Abcam, Cambridge, MA, USA). Detailed description of the methods for measuring oxidative stress markers is shown in Supplementary Methods.

### 2.8. IL-1*β* ELISA

The IL-1*β* level in the peripheral blood in rats after being exposed to PQ for 24 h was measured via ELISA assays using commercially available kits (R&D System, USA) according to the manufacturer's protocols. Standard curves were generated to extrapolate IL-1*β* levels in the samples.

### 2.9. Immunohistochemistry Staining

Paraffin-embedded liver sections were prepared according to routine methods mentioned above. Then, after incubating at 60°C for 1 h, these sections were deparaffinized, rehydrated, and washed in 0.01 M of citrate buffer. After inhibiting endogenous peroxidase using 3% H_2_O_2_ in methanol, the sections were incubated with anti-SIRT3 (Bioss, Beijing, China), anti-caspase-1 (Santa Cruz, CA, USA), anti-Nrf2, and anti-NLRP3 antibodies (Abcam, Cambridge, MA, USA) overnight at 4°C according to the instructions of Histostain™-Plus and DAB Substrate Kits. After being washed with a phosphate-buffered saline (PBS) solution, the sections were incubated with the corresponding secondary antibodies at room temperature for 30 min. These sections were then washed with TBS three times and incubated for 4-7 min in a solution of 0.02% diaminobenzidine containing 0.01% H_2_O_2_. Incubation with the secondary antibodies alone was used as a negative control. After counterstaining with hematoxylin, the images were captured using light microscopy (Nikon, Japan).

### 2.10. Cell Culture

The rat liver cell line BRL-3A was purchased from the Cell Bank of Type Culture Collection of Chinese Academy of Sciences (Shanghai, China) and cultured in DMEM (Gibco, USA) supplemented with 10% fetal bovine serum (FBS, HyClone, USA) at 37°C with 5% CO_2_. When the cells were 70%-80% confluent, the medium was removed and the cells were passaged. The medium was replaced every 2 days.

### 2.11. MTT Assay for Mitochondrial Activity

An MTT assay kit (Nanjing Jiancheng Corp., Nanjing, China) was used to evaluate the mitochondrial activity based on the principle that the water-soluble dye MTT (3-(4,5-dimethylthiazol-2-yl)-2,5-diphenyltetrazolium bromide) can be catalyzed by mitochondrial succinate dehydrogenase and transformed into an insoluble formazan in the metabolically active cells. The MTT assay indirectly serves to assess mitochondrial respiration and cellular energy capacity [37]. In brief, rat hepatocytes were seeded into 96-well plates at a density of 1 × 10^4^ cells per well in 100 *μ*L. After 12 h incubation, the cells were exposed to different concentrations (0, 10, 25, 50, 100, and 250 *μ*M) of PQ. After incubation with the indicated concentrations of PQ for 24 h, 10 *μ*L of 0.5 mg/mL MTT was added to each well and incubated for another 4 h. 150 *μ*L of DMSO was added after the supernatant was discarded. Finally, the absorbance at 570 nm was measured using a microplate reader (BioTek Instruments Inc., VT, USA).

### 2.12. Cell Treatment

Rat liver cells (1 × 10^5^ cells/mL) in 2.5 mL medium cultured in 6-well plates were seeded. After 12 h incubation, the cells were treated with PQ at the dose of 50 *μ*M based on the MTT assay results. To detect the protection of H_2_S against PQ-induced toxicity, the cells were pretreated with NaHS (50 *μ*M) [38, 39] for 30 min, washed twice with PBS, and then exposed to PQ. After exposure to PQ for 24 h, the cells were collected for molecular analysis.

### 2.13. Coimmunoprecipitation

The rat hepatocyte lysates (prepared as for immunoblots) were precleared with Protein G Magnetic SureBeads (Bio-Rad Laboratories, Hercules, CA). The protein (50 *μ*g) was immunoprecipitated by adding antibodies specific to Keap1 (Santa Cruz, CA, USA) or normal rabbit IgG and rotated for 15 min at room temperature. Immune complexes were then precipitated with protein G SureBeads. The bound proteins were then eluted by incubation with 4× SDS-PAGE sample buffer for 30 min at 37°C and analyzed by Western blotting with an anti-Nrf2 antibody (Abcam, Cambridge, MA, USA).

### 2.14. IDH2 Activity Analysis

Because IDH2 is an NADP^+^-dependent enzyme located in mitochondria, the mitochondria extraction kit (Sigma-Aldrich, St. Louis, MO, USA) was used to obtain mitochondrial homogenates according to the manufacturer's protocol. Briefly, the collected cells (1 × 10^6^) were harvested by centrifugation at 800 × *g* for 5 min and resuspended in lysis buffer after washing with PBS. The cells were homogenized using a Dounce homogenizer on ice, and the cell lysate was subsequently centrifuged at 1000 × *g* for 10 min to remove the nuclei and cell debris. After the supernatants were further centrifuged at 13000 × *g* for 10 min at 4°C, the mitochondrial pellets were resuspended in IDH assay buffer. Subsequently, the IDH2 activity was spectrophotometrically assayed using the IDH activity assay kit (Sigma-Aldrich, St. Louis, MO, USA) with a microplate reader (BioTek Instruments Inc., VT, USA). The absorbance at 450 nm was measured, and the enzyme activity of IDH2 was calculated for each sample according to the manufacturer's instructions. The values were normalized to the controls.

### 2.15. S-Sulfhydration Assay

Cells were homogenized in HEN buffer (1 mM EDTA, 0.1 mM neocuproine, and 250 mM HEPES-NaOH (pH 7.7)) supplemented with 100 *μ*M deferoxamine. After being centrifuged at 13000 × *g* for 30 min at 4°C, the cell lysates were added to blocking buffer (HEN buffer adjusted to 2.5% SDS (*w*/*v*) and 20 mM MMTS (methyl methanethiosulfonate)) at 50°C for 20 min with vortexing. After the MMTS removal by acetone, the proteins were precipitated for 20 min at -20°C. Following removal of the supernatant, 4 mM biotin-HPDP (biotin-N-[6-(Biotinamido)hexyl]-3′-(2-pyridyldithio) propionamide) in dimethyl sulfoxide (DMSO) without ascorbic acid in a HENS buffer containing 1% SDS (*w*/*v*) was added to the precipitated proteins. The biotinylated proteins were precipitated by streptavidin-agarose beads after incubation for 3 h at room temperature. After washing with HENS buffer, the biotinylated proteins were resolved by SDS-PAGE and subjected to Western blot analysis.

### 2.16. Nrf2 RNA Interference

To reduce Nrf2 mRNA levels, siRNAs against Nrf2 (Life Technologies, USA) were introduced. The sequences of the two siRNAs were as follows: siRNA1, 5′-GCA UGU UAC GUG AUG AGG AUG GAA A-3′, and siRNA2, 5′-UUC UGU CGC UGA CUA AAG UCA AAC A-3′. Rat hepatocytes were seeded in 12-well plates and at 50%-60% confluence and transfected with 100 nM siRNA with Lipofectamine 3000 in Opti-MEM (Invitrogen, USA) according to the manufacturer's suggested instructions. The transfection with scrambled siRNA was used as a negative control. The cells were incubated at 37°C in a CO_2_ incubator for 24 h. After transfection, the cells transfected with siRNA were exposed to PQ (50 *μ*M) with or without NaHS (50 *μ*M) and the protein was then collected and assessed by Western blot analysis.

### 2.17. Western Blot Analysis

Liver tissue homogenates or hepatocytes were placed on ice and lysed with ice-cold NDET buffer (150 mM NaCl, 1% NP-40, 0.4% deoxycholic acid, 5 mM EDTA, and 25 mM Tris (pH 7.4)) supplemented with complete protease inhibitor cocktail (Roche Applied Science, Indianapolis, USA) for 15 min. Lysates were centrifuged at 18000 × *g* for 10 min at 4°C to remove nuclei and insoluble debris. The nuclear proteins were extracted using commercially available kits (Nanjing Jiancheng Corp., Nanjing, China) according to the manufacturer's recommended protocol. The protein concentrations were determined by the Bio-Rad Protein Assay (Bio-Rad, Hercules, CA, USA). Samples (50 *μ*g) were separated on 10% SDS-PAGE and transferred to PVDF (Polyvinylidene Difluoride) membranes. The membranes were blocked for 1 h with TBS Tween (TBST) containing 10% nonfat milk at room temperature after washing. Then, the membranes were incubated with primary antibodies against Keap1, Nrf2, IDH2, NLRP3 (Abcam, Cambridge, MA, USA), SIRT3 (Bioss, Beijing, China), caspase-1 (Santa Cruz, CA, USA), and IL-1*β* (R&D, USA) at 4°C overnight with consistent agitation. The antibodies of *β*-actin, glyceraldehyde-3-phosphate dehydrogenase (GAPDH), voltage-dependent anion-selective channel 1 (VDAC1), and TATA-box-binding protein (TBP) (Abcam, Cambridge, MA, USA) were used for loading controls. After washing three times with TBST, the membranes were incubated with appropriate secondary antibodies at room temperature for 2 h. The proteins were then detected using the enhanced chemiluminescence detection kit according to the manufacturer's instructions. Intensities of the immunoreactive bands were quantified using ImageJ software (National Institutes of Health (NIH), Bethesda, MA, USA).

### 2.18. Statistical Analysis

All data analyses were carried out using SPSS 19.0 (SPSS Inc., Chicago, IL, USA). Results were shown as the mean ± SEM of at least three independent experiments. Comparisons among multiple groups were performed with one-way analysis of variance (ANOVA) followed by the Student-Newman-Keuls test. Comparisons between two groups were determined by Student's *t*-test. A *P* value of <0.05 defined statistical significance.

## 3. Results

### 3.1. Exogenous H_2_S Attenuated PQ-Induced Liver Injury and Elevation of Hepatic Aminotransferase Levels in Serum

**Figure 1:**
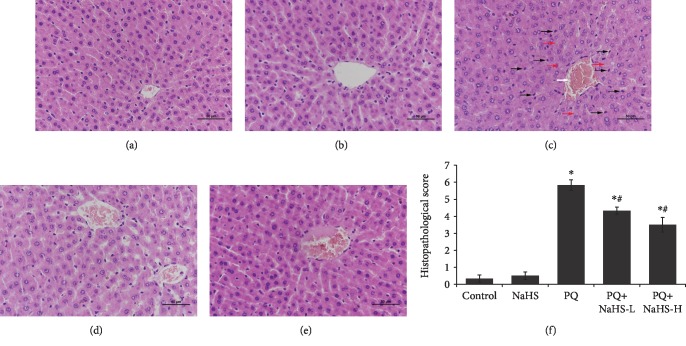
Effects of exogenous H_2_S on histopathological changes in the rat liver after PQ administration. H&E staining was performed to examine histopathological change of the rat liver after PQ (20 mg/kg) administration for 24 h. Histological appearance of the liver sections was studied using a light microscope. Red arrows indicate cellular ballooning; black arrows indicate infiltration of inflammatory cells; the white arrow indicates central venous congestion. (a) Control group. (b) NaHS group. (c) PQ group. (d) PQ+NaHS-L group (3 mg/kg of NaHS, i.p.). (e) PQ+NaHS-H group (5 mg/kg of NaHS, i.p.). (f) Histopathological score. Data are expressed as means ± SEM (*n* = 6/group). ^∗^*P* < 0.05, compared with control group; ^#^*P* < 0.05, compared with PQ group.

**Figure 2:**
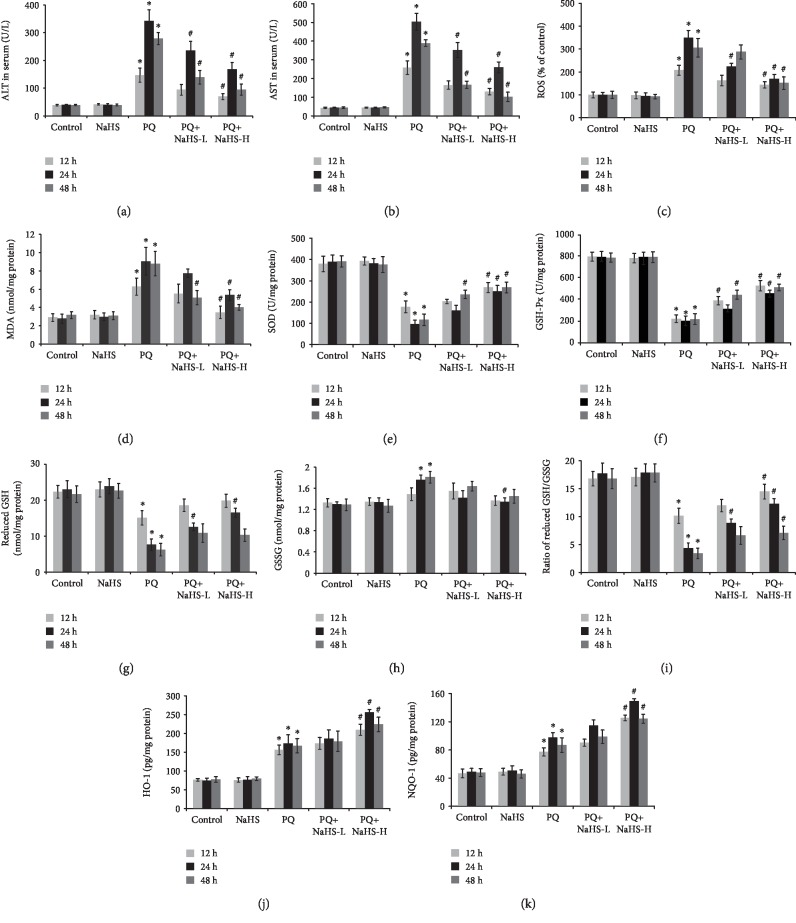
Effects of exogenous H_2_S on the aminotransferases and oxidative stress marker in the rat liver after PQ administration. The rat liver samples were collected following PQ (20 mg/kg) administration for 12, 24, and 48 h to analyze hepatic aminotransferase levels and markers of oxidative stress. (a) ALT levels. (b) AST levels. (c) ROS generation. (d) MDA content. (e) SOD activity. (f) GSH-Px activity. (g) Reduced GSH content. (h) GSSG content. (i) Ratio of reduced GSH/GSSG. (j) HO-1 levels. (k) NQO-1 levels. Data are expressed as means ± SEM (*n* = 6/group). ^∗^*P* < 0.05, compared with control group; ^#^*P* < 0.05, compared with PQ group.

As shown in Figure 1, microscopic examination of liver tissue in the PQ poisoning group revealed cellular damages, severe cellular ballooning, infiltration of inflammatory cells, and central venous congestion. Exogenous H_2_S attenuated the PQ-induced hepatic damage, particularly at the high dose of NaHS (5 mg/kg). In addition, the serum levels of hepatic aminotransferases including ALT and AST were significantly increased in the PQ-intoxicated rats with levels peaking at the time point of 24 h. Exogenous H_2_S at least partly diminished the observed increase in ALT and AST levels in serum from PQ-intoxicated rats (Figures 2(a) and 2(b)).

### 3.2. Exogenous H_2_S Attenuated PQ-Induced Oxidative Stress

**Figure 3:**
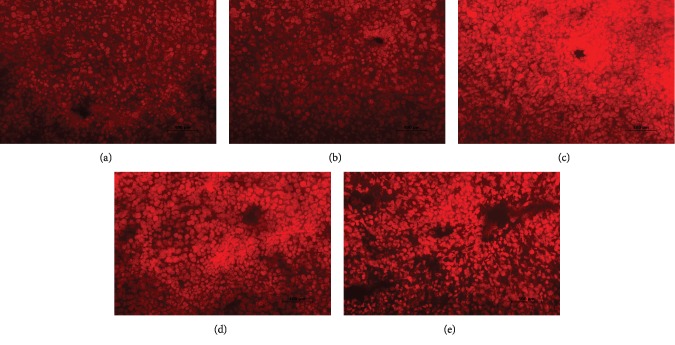
Effects of exogenous H_2_S on the ROS generation in the rat liver after PQ administration. DHE staining was performed to measure ROS generation in the rat liver tissue after PQ (20 mg/kg) administration for 24 h. (a) Control group. (b) NaHS group. (c) PQ group. (d) PQ+NaHS-L group (3 mg/kg of NaHS, i.p.). (e) PQ+NaHS-H group (5 mg/kg of NaHS, i.p.).

As shown in Figures 3 and 2, PQ intoxication in rats significantly increased ROS production and MDA (membrane lipid peroxidation end product) content in the liver tissue, which were suppressed by NaHS. PQ intoxication impaired the activities of the antioxidant enzymes including SOD, GSH-Px, HO-1, and NQO-1 in the liver tissue, which were enhanced by NaHS. Additionally, PQ intoxication significantly altered the expression of GSH and GSSG in the liver tissue reducing the ratio of GSH/GSSG, which was attenuated by NaHS. The data indicates that H_2_S exerted a protective effect on PQ-induced oxidative stress.

### 3.3. Exogenous H_2_S Induced Keap1 S-Sulfhydration and Nuclear Translocation of Nrf2

**Figure 4:**
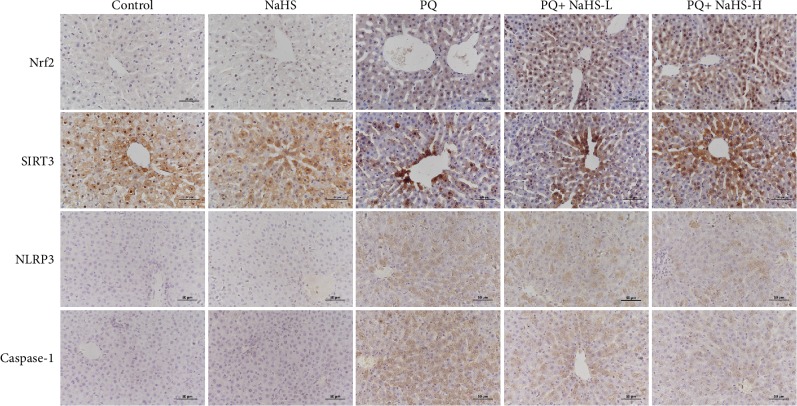
Effects of exogenous H_2_S on the expression of Nrf2, SIRT3, NLRP3, and caspase-1 in the rat liver after PQ administration. Immunohistochemistry staining was performed to determine the expression of Nrf2, SIRT3, NLRP3, and caspase-1 in liver tissue after PQ administration for 24 h. The positive signals of Nrf2, SIRT3, NLRP3, and caspase-1 were shown in the PQ group. In contrast, NaHS attenuated the expression of these proteins in the rat liver.

**Figure 5:**
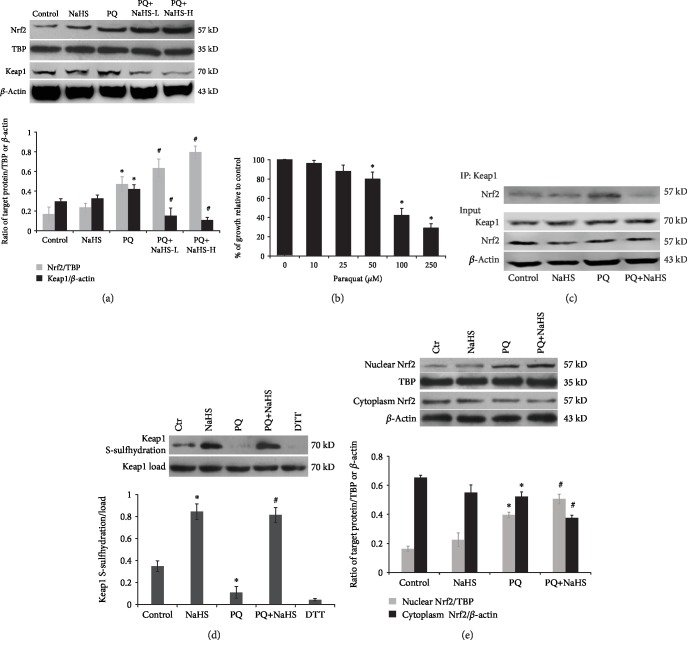
Effects of exogenous H_2_S on Keap1/Nrf2 signaling in PQ-induced acute liver injury. (a) Western blot analysis was performed to detect the expression of Keap1 and Nrf2 in the rat liver after PQ administration for 24 h (*n* = 6/group). (b) Mitochondrial activity was measured by the MTT assay at 24 h after rat hepatocytes were exposed to the indicated concentrations of PQ (*n* = 3). (c) The rat hepatocytes were exposed to PQ (50 *μ*M) with or without NaHS (50 *μ*M). Cell lysates were immunoprecipitated with an anti-Keap1 or an anti-IgG antibody (negative control) and blotted with an anti-Nrf2 antibody (top panel). (d) NaHS stimulated Keap1 S-sulfhydration in rat hepatocytes, and the effect was reversed by dithiothreitol (DTT) (*n* = 3). (e) Western blot analysis was used to detect the expression of Nrf2 in rat hepatocytes exposed to PQ (50 *μ*M) for 24 h with or without NaHS (50 *μ*M) (*n* = 3). Data are expressed as means ± SEM. ^∗^*P* < 0.05, compared with control group; ^#^*P* < 0.05, compared with PQ group.

Nrf2 is a key regulator for genes encoding antioxidant enzymes including SOD, HO-1, and NQO-1 against oxidative stress [40]. Keap1 binds to Nrf2 in the cytoplasm inhibiting Nrf2 activation during unstressed conditions. As shown in Figures 4 and 5(a), PQ slightly induced the expression of Nrf2 and Keap1. Furthermore, the accumulation of Nrf2 in the nucleus was significantly increased in the PQ-poisoned rats treated with NaHS. In *in vitro* studies, the results shown in Figures 5(c)–5(e) suggest that ROS generated by PQ promoted the reduction of Keap1 S-sulfhydration, which resulted in Nrf2 dissociation from Keap1, and enhanced Nrf2 nuclear translocation. NaHS alone enhanced Keap1 S-sulfhydration without Nrf2 nuclear translocation under the unstressed condition. Following exposure to PQ, NaHS increased Keap1 S-sulfhydration and nuclear Nrf2 expression in hepatocytes. The nuclear translocation of Nrf2 enhanced by exogenous H_2_S exerted protective effects against PQ-induced acute liver injury via S-sulfhydration of Keap1.

### 3.4. Exogenous H_2_S Reversed the Reduction of SIRT3/IDH2 Induced by PQ

**Figure 6:**
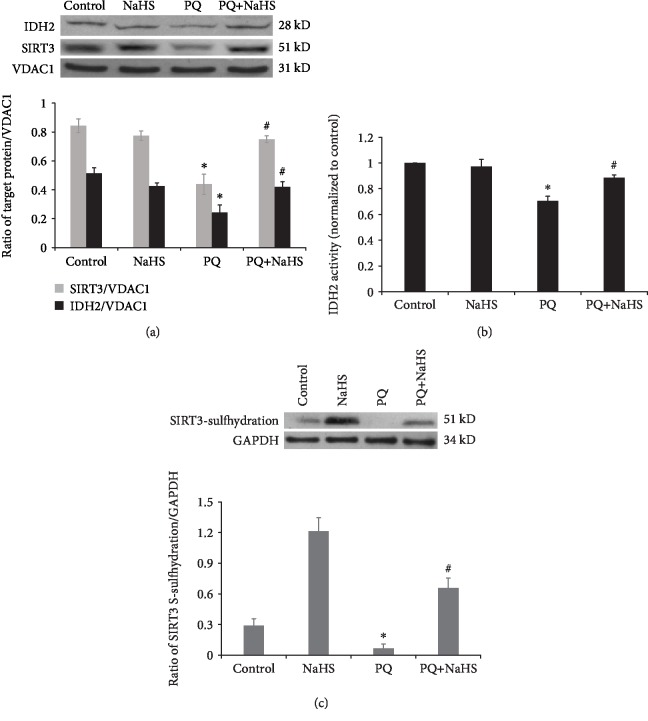
Effects of exogenous H_2_S on SIRT3/IDH2 signaling in PQ-induced hepatotoxicity. (a) Western blot analysis was used to detect the expression of mitochondrial SIRT3 and IDH2 in rat hepatocytes exposed to PQ (50 *μ*M) for 24 h with or without NaHS (50 *μ*M) (*n* = 3). (b) NaHS attenuated the decrease in IDH2 activity induced by PQ in rat hepatocytes (*n* = 3). (c) NaHS enhanced SIRT3 S-sulfhydration in rat hepatocytes (*n* = 3). Data are expressed as means ± SEM. ^∗^*P* < 0.05, compared with control group; ^#^*P* < 0.05, compared with PQ group.

Isocitrate dehydrogenase-2 (IDH2) which can catalyze the irreversible oxidation and decarboxylation of isocitrate into *α*-ketoglutarate is partly regulated by SIRT3, the primary mitochondrial deacetylase. In this study, the expression of SIRT3 in the cytoplasm was reduced substantially in PQ-intoxicated rats, but treatment with NaHS prevented the observed reduction in SIRT3 expression (Figures 4 and 6). Furthermore, exposure to PQ significantly decreased the expression of SIRT3 and IDH2, as well as the activity of IDH2 in rat hepatocytes (*P* < 0.05), which was remarkedly reversed by the addition of NaHS (Figures 6(a) and 6(b)). Simultaneously, exogenous H_2_S may regulate SIRT3/IDH2 signaling by S-sulfhydration of SIRT3 (Figure 6(c)).

### 3.5. Exogenous H_2_S Inhibited PQ-Induced NLRP3 Inflammasome Activation

**Figure 7:**
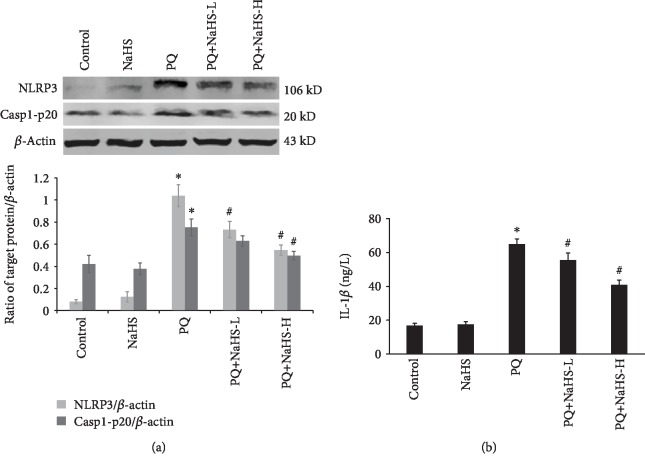
Effects of exogenous H_2_S on NLRP3 inflammasome activation in PQ-induced acute liver injury. (a) Western blot analysis was performed to detect the expression of NLRP3 and caspase-1-p20 in the rat liver after PQ administration for 24 h (*n* = 6/group). (b) The IL-1*β* levels in the peripheral blood in rats after being exposed to PQ for 24 h were measured with ELISA (*n* = 6/group). Data are expressed as means ± SEM. ^∗^*P* < 0.05, compared with control group; ^#^*P* < 0.05, compared with PQ group.

Oxidative stress may trigger NLRP3 inflammasome activation and subsequent IL-1*β* secretion [41, 42]. In this study, PQ administration induced NLRP3 protein expression in the rat liver (Figure 4), and this effect could be significantly inhibited by NaHS pretreatment. Caspase-1 activation mediated by the NLRP3 inflammasome is a key factor in a variety of inflammatory diseases. As shown in Figure 7, the caspase-1 cleavage and IL-1*β* secretion were observed in the PQ-intoxicated rats and the proinflammatory effect was significantly attenuated by NaHS. Herein, exogenous H_2_S inhibited NLRP3 inflammasome activation and IL-1*β* secretion in PQ-induced liver injury.

### 3.6. Nrf2 Knockdown Abolished the Protective Effect of H_2_S against PQ-Induced SIRT3/IDH2 and NLRP3 Inflammasome Activation

**Figure 8:**
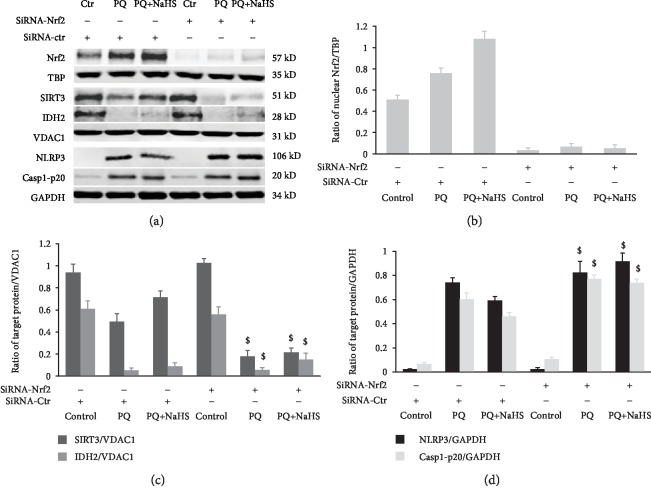
Role of Nrf2 in the protective effect of H_2_S against PQ-induced hepatotoxicity. Rat hepatocytes were pretreated with siRNA against Nrf2 24 h before exposure to PQ (50 *μ*M). Western blot analysis was used to detect the expression of SIRT3, IDH2, NLRP3, and caspase-1-p20. Data are expressed as means ± SEM. ^$^*P* < 0.05, compared with the corresponding control group pretreated with siRNA-Nrf2.

As shown in Figure 8, the siRNA targeting Nrf2 significantly suppressed the expression of Nrf2 in rat hepatocytes. NaHS attenuated the effects of PQ resulting in the increased expression of SIRT3/IDH2 and NLRP3, as well as caspase-1 cleavage. Interestingly, the decrease in SIRT3/IDH2 caused by PQ was augmented after siRNA-Nrf2 pretreatment; NLRP3 inflammasome activation induced by PQ was further increased by siRNA-Nrf2 pretreatment. NaHS did not exert a protective effect against PQ-induced hepatotoxicity following siRNA-Nrf2 pretreatment. Therefore, downregulation of Nrf2 may abolish the protective effect of exogenous H_2_S against PQ-induced hepatotoxicity.

## 4. Discussion

The liver is regarded as one of the major targets of paraquat [43]. In this study, PQ induced various histological changes in the rat liver, including severe cellular ballooning, central venous congestion, infiltration of inflammatory cells, and loss of cell boundaries. These histopathological findings supported by elevated serum ALT and AST levels after PQ administration agreed with previous reports [3, 4, 43]. As an electron acceptor, PQ donates an electron to oxygen and superoxide anion (O_2_^−^) and its derivatives (i.e., H_2_O_2_ and ^·^OH) are then generated [44]. As an initiating factor, oxidative stress plays a key role in triggering PQ-induced liver injury [45]. The redox reaction of PQ causes depletion of NADPH and GSH, mitochondrial dysfunction, and subsequent lipid peroxidation of cellular membranes [2]. As a typical marker of lipid peroxidation, the level of MDA in liver tissue was significantly increased after PQ exposure. In addition, SOD, GSH-Px, HO-1, and NQO-1 function as critical protective enzymes against oxidative stress and regulate the cellular concentration of ROS. These enzymes prevent the negative effects of peroxide radicals and the initiation of lipid peroxidation [4, 46]. Recent studies demonstrate HO-1 and NQO-1 as having the capability to attenuate PQ-induced toxicity [47, 48]. In agreement, the data suggests that antioxidant enzymes including SOD, GSH-Px, HO-1, and NQO-1 were, at least in part, involved in PQ-induced liver injury.

As a thiol-containing peptide, *γ*-Glu-Cys-Gly, GSH, is synthesized in all mammalian cells, being particularly abundant in the liver. GSH is a critical antioxidant in maintaining the cellular redox balance [49]. GSH contributes to free radical scavenging through the interaction with GSH S-transferase directly or via a reaction catalyzed by GSH-Px, in which two identical molecules of GSH are oxidized to form GSH disulfide (GSSG) [50]. GSH content is decreased following exposure to oxidants, and the knockdown of GSH renders cells more susceptible to oxidants, such as paraquat [51]. GSH-Px, an enzyme which can catalyze GSH to form GSSG to alleviate oxidative stress, removes harmful peroxide metabolites and inhibits lipid peroxidation [52]. Consistent with previous reports [53, 54] where PQ decreased GSH levels and enhanced GSSG levels, the ratio of GSH/GSSG and GSH-Px activity were reduced in rat liver tissue in this study.

H_2_S is synthesized from L-cysteine by three different enzymes including cystathionine-*β*-synthase (CBS), cystathionine-*γ*-lyase (CSE), and 3-mercaptopyruvate sulfurtransferase (3-MST) that control the spatial and temporal formation of H_2_S in the body [55]. Additionally, H_2_S is also generated by the metabolic activity of resident gut microbes, mainly by colonic Sulfate-Reducing Bacteria (SRB) via the enzyme complex dissimilatory sulfite reduction system including ATP sulfurylase (ATPS), adenosine-5-phosphosulfate (APS) reductase, and dissimilatory sulfite reductase (DSR) [56]. H_2_S exerts protective effects regarding ROS-mediated damage of membranes by elevating the GSH production via activation of cysteine/cystine transporters or maintenance of the cellular GSH redox status. Moreover, H_2_S may enhance GSH transport into mitochondria and promote redistribution of GSH [55, 57]. H_2_S can regulate redox reaction, mitochondrial function, and inflammatory signal transduction [58]. Exogenous H_2_S exerts protective effects against liver ischemia-reperfusion (I/R) injury [59]. In this study, exogenous H_2_S attenuated liver pathological alterations and the increased levels of both serum AST and ALT in PQ-intoxicated rats. Exogenous H_2_S inhibited the increase in ROS and MDA levels induced by PQ. Additionally, exogenous H_2_S not only enhanced the activity of antioxidants including SOD, HO-1, and NQO-1 in PQ-intoxicated rats but also increased the hepatic levels of GSH and GSH-Px, as well as the ratio of GSH/GSSG. Elevation of GSH content in the liver is partly associated with the beneficial effects of exogenous H_2_S in PQ-induced hepatotoxicity. Taken together, the protective effect of H_2_S against oxidative stress is mediated by its ability to directly scavenge ROS, degrade lipid peroxides, upregulate GSH synthesis, and enhance antioxidant activity [60].

The Keap1/Nrf2 pathway plays an important role in response to oxidative stress. Keap1 acts as a cysteine thiol-rich sensor, whereas Nrf2 is a transcription factor regulating the synthesis of antioxidant enzyme genes. Currently, a widely accepted theory for Nrf2 nuclear translocation during oxidative stress is that modification of Keap1 cysteines leads directly to dissociation of the Keap1-Nrf2 complex [61]. Besides the regulation of Nrf2 expression and its interaction with Keap1, posttranslational modification of Nrf2 (e.g., protein kinase C-dependent phosphorylation) may also result in changes in Nrf2 activity and/or localization [62, 63]. S-Sulfhydration, the addition of one sulfhydryl to the thiol side of the cysteine residue and formation of a persulfide group (R-S-S-H), is a critical posttranslational modification (PTM) in H_2_S signaling [64]. NaHS can S-sulfhydrate Keap1 at cysteine 151, induce Nrf2 dissociation from Keap1, enhance Nrf2 nuclear translocation, and stimulate mRNA expression of Nrf2-targeted downstream genes including GSH reductase and glutamate-cysteine ligase [65]. In this study, PQ exposure contributed to the dissociation of Nrf2 from Keap1 in the cytoplasm and translocation of Nrf2 to the nucleus. Exogenous H_2_S S-sulfhydrated Keap1, further triggered Nrf2 nuclear accumulation, and enhanced the expression of downstream cytoprotective enzymes such as HO-1, NQO-1, and SOD in PQ-intoxicated hepatocytes. Nevertheless, it is worth noting that H_2_S exerts beneficial as well as deleterious effects [66]. Low concentrations of NaHS stimulate the growth of hepatocellular carcinoma cells, whereas greater concentrations of NaHS exert an inhibitory effect [66]. Exposure to high concentration of NaHS (0.5 mM) could induce hepatocyte toxicity and rapid cell necrosis by inhibiting cytochrome oxidase in addition to generating ROS [67]. H_2_S exerts either an injurious or protective effect on hepatocytes through a mechanism likely associated with the relative concentration of H_2_S. Considering the prooxidative and antioxidative effects of H_2_S at various doses [67, 68], the relatively low doses of NaHS were used to investigate the antioxidative effects of NaHS against the PQ-induced liver injury. The rat hepatocytes were pretreated with NaHS at a dose of 50 *μ*M; likewise, the rats were pretreated with NaHS at a dose of 3 or 5 mg/kg in this study. Compared with the control group, ROS levels were not significantly increased in rats treated with NaHS alone (Figures 3 and 2) and Nrf2 nuclear translocation or the increase in SOD, HO-1, and NQO-1 expression was not observed (Figures 2 and 5). These findings above are consistent with previous studies [34, 35, 38, 39].

SIRT3 is a histone deacetylase localized predominantly in mitochondria and regulates mitochondrial enzymatic activity in the tricarboxylic acid (TCA) cycle [69]. One substrate in the TCA cycle, IDH2, at least in some significant part, is deacetylated by SIRT3 [14, 70]. IDH2 is a major producer of mitochondrial NADPH, required for the GSH-associated mitochondrial antioxidant systems [71]. Exogenous H_2_S restores the expression of Nicotinamide Phosphoribosyltransferase (NAMPT) which suppresses oxidative stress by increasing NADPH levels and enhances the expression and activity of SIRT3 [64, 72]. As a result of activation of SIRT3, acetylation of the respiratory complex enzyme NADH dehydrogenase 1 (ND1) and glucose oxidation enzymes including pyruvate dehydrogenase (PDH), citrate synthase (CS), and IDH2 is reduced, which can enhance the activities of the mitochondrial respiratory chain activity and result in the activation of PDH, IDH2, and CS [64, 73]. H_2_S protects much larger ranges of redox-sensitive proteins including PDH, IDH2, and CS to attenuate oxidative stress. In this study, exogenous H_2_S reversed the effect of PQ reducing the expression of SIRT3 and IDH2, as well as IDH2 activity in hepatocytes. Furthermore, S-sulfhydration of SIRT3 induced by H_2_S promoted the expression and activity of IDH2. H_2_S exerted a protective effect against PQ-induced mitochondrial dysfunction via the SIRT3/IDH2 pathway. These findings are consistent with the recent finding that H_2_S attenuated cisplatin-induced acute kidney injury by preventing mitochondrial dysfunction via SIRT3 S-sulfhydration [74]. Nevertheless, it should be noted that the mechanisms of mitochondrial protection by H_2_S are not only dependent on SIRT3/IDH2 as the protective effect is also mediated by SQR (sulfur quinone reductase) [75], the electron transport chain, and ATP synthesis [64].

Oxidative stress is considered an important driver of NLRP3 inflammasome activation, and ROS scavenging can attenuate NLRP3 inflammasome activation [76]. Our previous studies indicate that the ROS-mediated NLRP3 inflammasome is involved in PQ-induced lung and kidney injury [18, 19, 77]. In this study, the data shows that PQ administration caused NLRP3 inflammasome activation and proinflammatory factor IL-1*β* expression in the rat liver. Notably, the great number of leukocytes that were recruited into the hepatic tissue in response to PQ is clearly visible (Figure 1), which in turn can significantly contribute to the increased production of ROS. It should be noted that the oxidative liver modifications induced by PQ were mediated by dysfunctional hepatocyte mitochondria in addition to activated leukocytes that have migrated into the tissue. H_2_S reduced NLRP3 inflammasome activation while diminishing mitochondrial ROS production by promoting antioxidant activity in PQ-induced liver injury. This is in line with the studies showing that H_2_S attenuated NLRP3 inflammasome activation induced by oxidative stress or dextran sulfate sodium (DSS) [24, 78]. It is worth mentioning that DSS-induced colitis was highly dependent on leukocyte migration and recruitment into the colonic tissue [24]. Experimental evidence indicates that H_2_S attenuates the mitochondrial ROS production and NLRP3 inflammasome activation via S-sulfhydration of c-Jun (subunit of activator protein-1 (AP-1)) at cysteine 269 in macrophages [78]. Therefore, NLRP3 inflammasome activation inhibited by H_2_S in vivo is a complex process, involving inflammatory cell infiltration and cytokine release.

As a transcription factor, Nrf2 plays a crucial role in the expression of the mitochondrial gene SIRT3 [79]. Nrf2 can bind to the SIRT3 promoter regulating SIRT3 expression. When cells are exposed to oxidative stress, nuclear translocation of Nrf2 can promote SIRT3 upregulation [79, 80]. SIRT3 reduces the level of mtROS and enhances mitochondrial ROS-scavenging capacity by deacetylation of the major mitochondrial antioxidant enzymes, including SOD2, IDH2, and GSH-Px [14, 81]. Nrf2 is identified as an inhibitor of NLRP3 expression at the transcriptional level. Nrf2 negatively regulates NLRP3 inflammasome activity and subsequent IL-1*β* generation by inhibiting ROS-induced NLRP3 transcription [82]. Moreover, NQO-1 is involved in the negative regulation of Nrf2 on NLRP3 inflammasome activation [82]. In this study, H_2_S cannot reverse the decrease in SIRT3 and activation of the NLRP3 inflammasome caused by PQ in Nrf2-knockdown hepatocytes. Therefore, H_2_S cannot exert a protective effect against PQ-induced hepatotoxicity following Nrf2 knockdown. Nrf2 plays a vital role in the protective effect of H_2_S against acute liver injury induced by PQ.

**Figure 9:**
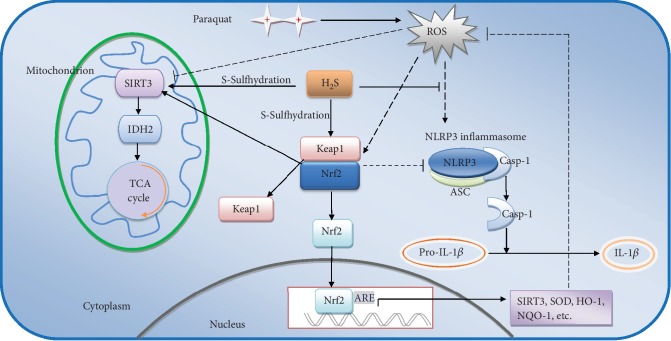
The potential molecular mechanism by which H_2_S ameliorates PQ-induced acute liver injury.

In conclusion, H_2_S ameliorates the toxic effects of PQ on the liver by enhancing antioxidative capability, regulating mitochondrial function, and suppressing ROS-induced NLRP3 inflammasome activation. The antioxidative effect of H_2_S can at least partly be attributed to Nrf2 nuclear translocation via Keap1 S-sulfhydration and regulation of SIRT3/IDH2 signaling via Nrf2-dependent SIRT3 gene transcription as well as SIRT3 S-sulfhydration (Figure 9). Therefore, exogenous H_2_S administration may be of therapeutic benefit in PQ-induced acute liver injury.

## Data Availability

The data used to support the findings of this study are available from the corresponding author upon request.

## Abbreviations

ALT: Alanine aminotransferase
AST: Aspartate aminotransferase
DTT: Dithiothreitol
GSH: Reduced glutathione
GSH-Px: Glutathione peroxidase
H_2_S: Hydrogen sulfide
HO-1: Heme oxygenase-1
HPDP: N-[6-(Biotinamido)hexyl]-3′-(2-pyridyldithio) propionamide
IL-1*β*: Interleukin-1*β*
IDH2: Isocitrate dehydrogenase 2
Keap1: Kelch-like ECH-associated protein 1
MDA: Malondialdehyde
MTT: Methylthiazolyldiphenyl-tetrazolium bromide
NADPH: Nicotinamide adenine dinucleotide phosphate
NLRP3: Nucleotide-binding domain and leucine-rich repeat containing protein 3
NQO-1, NAD(P)H: Quinone oxidoreductase-1
Nrf2: Nuclear factor erythroid-2-related factor 2
PQ: Paraquat
ROS: Reactive oxygen species
SIRT3: Sirtuin 3
SOD: Superoxide dismutase
TCA: Tricarboxylic acid
VDAC-1: Voltage-dependent anion-selective channel 1.

## Supplementary Materials

Supplementary Materials Measurement of oxidative stress markers. The detailed protocols for the measurement of oxidative stress markers including malondialdehyde (MDA) contents, superoxide dismutase (SOD) activity, glutathione peroxidase (GSH-Px) activity, reduced glutathione (GSH) content, and heme oxygenase 1 (HO-1) and NAD(P)H: quinone oxidoreductase 1 (NQO-1) contents in rat liver tissues were shown.
