# Supplementary material for: Hydrogen Sulfide Protects against Paraquat-Induced Acute Liver Injury in Rats by Regulating Oxidative Stress, Mitochondrial Function, and Inflammation
Source: Oxid Med Cell Longev. 2020 Jan 23;2020:6325378. doi: 10.1155/2020/6325378 (PMC6998754; doi:10.1155/2020/6325378)
Supplement: Supplementary Materials — Measurement of oxidative stress markers. The detailed protocols for the measurement of oxidative stress markers including malondialdehyde (MDA) contents, superoxide dismutase (SOD) activity, glutathione peroxidase (GSH-Px) activity, reduced glutathione (GSH) content, and heme oxygenase 1 (HO-1) and NAD(P)H: quinone oxidoreductase 1 (NQO-1) contents in rat liver tissues were shown. [file 6325378.f1.docx]

**Supplementary methods**

**Measurement of Oxidative Stress Markers**

The detailed protocols for the measurement of oxidative stress markers including malondialdehyde (MDA) content, superoxide dismutase (SOD) activity, glutathione peroxidase (GSH-Px) activity, reduced glutathione (GSH)/oxidized GSH (GSSG) content, heme oxygenase 1 (HO-1) and NAD(P)H: quinone oxidoreductase 1 (NQO-1) content in rat liver tissues are as follows:

***1. Measurement of MDA content***

The MDA content in liver tissue was determined using the MDA assay kit (Nanjing Jiancheng Corp., Nanjing, China) based on thiobarbituric acid (TBA) reactivity. In brief, after mixing trichloroacetic acid with the homogenate and centrifuging, TBA was added to the supernatants. The developed red color of the resulting reaction was measured by absorbance at 532 nm with a microplate reader (Bio-Tek Instruments Inc., VT, USA). The other procedures were carried out according to the manufacturer’s protocols.

***2. Measurement of SOD activity***

The activity of SOD was determined by measuring the inhibiting rate of the enzyme to $O_{2}^{-.}$ produced by the xanthine morpholine with xanthine oxidase using the SOD assay kit (Nanjing Jiancheng Corp., Nanjing, China). The red substances of the reaction system were detected and quantified by the absorbance at 550 nm with a microplate reader (Bio-Tek Instruments Inc., VT, USA) after 40 min of reaction time at 37℃. One unit of SOD activity (U) is defined as the quantity of SOD required to produce 50% inhibition of reduction of nitrite in 1 mL reaction solution by measuring the change of absorbance at 550 nm. SOD activity was calculated with the formula detailed in the manufacturer’s instructions.

***3. Measurement of GSH-Px activity***

GSH-Px activity was determined by the colorimetric method using the GSH-Px assay kit (Nanjing Jiancheng Corp., Nanjing, China) based on the principle that oxidation of glutathione (GSH) and hydrogen peroxide (H_2_O_2_) could be catalyzed by GSH-Px to produce oxidized glutathione (GSSG) and H_2_O. GSH reacts with 5, 5′-dithiobis (2-nitrobenzoic acid) (DTNB) to produce stable yellow substances which cause the change of absorbance at 412 nm detected with a microplate reader (Bio-Tek Instruments Inc., VT, USA). The decrease of GSH at 412 nm during the reaction is indicative of GSH-Px activity in liver tissues. According to the manufacturer’s instructions, one GSH-Px unit of GSH-Px activity (U) was calculated as the amount of enzyme that will oxidize 1 µmol/L GSH in a reaction system at 37℃ per minute in 1.0 g fresh tissue.

***4. Measurement of reduced GSH & oxidized GSH (GSSG) contents***

The concentrations of total GSH and oxidized GSH (GSSG) in liver tissues were determined by the colorimetric method using the Total-GSH/GSSH assay kit (Nanjing Jiancheng Corp., Nanjing, China) based on the principle that reduced GSH can react with 5, 5′-dithiobis (2-nitrobenzoic acid) (DTNB) to yield a stable yellow signal. Before the determination of GSSG, the “masking reagent” binding GSH was added to the samples and incubated in buffer solution for 30 min at 37℃. To detect the concentrations of total GSH and GSSG, the absorbance at 405 nm was measured with a microplate reader (Bio-Tek Instruments Inc., VT, USA) at two timepoints: 30 sec and 10.5 min after the reaction started at room temperature. The concentrations of total GSH and GSSG were calculated based on the manufacturer's formula. The concentration of reduced GSH was calculated as total GSH minus 2 × GSSG. The concentrations of GSH and GSSG were expressed as nmol/mg protein.

***5. Measurement of HO-1 & NQO-1 contents***

The HO-1 and NQO-1 contents were measured by ELISA using the HO-1 and NQO-1 assay kits (Abcam, Cambridge, MA, USA), respectively. According to the manufacturer's instructions, the absorbance indicating the relative expression levels of HO-1 and NQO-1 at 450 nm was measured using a microplate reader (Bio-Tek Instruments Inc., VT, USA). The concentrations of HO-1 or NQO-1 in the samples were determined by comparing the absorbance of the samples to the standard curve.
